# Researchers’ experiences working with community advisory boards: How community member feedback impacted the research

**DOI:** 10.1017/cts.2021.22

**Published:** 2021-03-17

**Authors:** Tabetha A. Brockman, Joyce E. Balls-Berry, Ian W. West, Miguel Valdez-Soto, Monica L. Albertie, Noreen A. Stephenson, Farhia M. Omar, Mitch Moore, Marty Alemán, Pastor Albert Berry, Suganya Karuppana, Christi A. Patten

**Affiliations:** 1 Center for Clinical and Translational Science, Community Engagement in Research Program, Mayo Clinic, Rochester, MN, USA; 2 Department of Psychiatry and Psychology, Mayo Clinic, Rochester, MN, USA; 3 Center for Health Equity and Community Engagement Research, Mayo Clinic, Rochester, MN, USA; 4 Behavioral Health Research Program, Mayo Clinic, Rochester, MN, USA; 5 Knight Alzheimer’s Disease Research Center Health Disparities and Equity Core, Washington University, St. Louis, MO, USA; 6 Counselor Education Department, Winona State University, Rochester, MN, USA; 7 Department of Nursing, Augsburg University, Rochester, MN, USA; 8 Faith Chapel Church of God, Jacksonville, FL, USA; 9 Department of Family Medicine, Adelante Healthcare, Phoenix, AZ, USA

**Keywords:** Community advisory board, community engagement, research

## Abstract

**Introduction:**

To assess researchers’ experiences working with community advisory boards (CABs) and perceptions of how community member stakeholder feedback impacted the research.

**Methods:**

Individual interviews were conducted with researchers (*n*= 34) who had presented their research to a Mayo Clinic CAB (at MN, AZ, or FL) from 2014 to 2017, with an average interview duration of 10–15 min. Researchers were asked “In what ways did the feedback you received from the CAB influence your research?” A validated, structured, 7-item interview was used to assess domains of the potential influence that CABs had on the research: (1) pre-research (e.g., generated ideas), (2) infrastructure (e.g., budget preparation), (3) research design, (4) implementation (e.g., research recruitment), (5) analysis, (6) dissemination, and (7) post-research. A total mean score was calculated with a possible range of 0–7. In addition, open-ended examples and feedback from researchers in response to each domain were summarized for themes using content analysis.

**Results:**

Researchers reported that the CAB influenced research in the following domains: pre-research (24%), infrastructure (24%), study design (41%), implementation (41%), analysis (6%), dissemination (24%), and post-research activities (18%). The mean total score was = 1.8 (SD = 1.7, range: 0–6). Open-ended responses revealed major themes of CAB helpfulness in generating/refining ideas, identifying community partners, culturally tailored and targeted recruitment strategies, intervention design and delivery, and dissemination.

**Conclusion:**

Findings from this preliminary evaluation indicate that despite positive experiences noted in open-ended feedback, the perceived quantitative impact of CAB feedback on the research was moderate. Bidirectional communication between researchers and community member stakeholders has the potential to make clinical and translational research more relevant and appropriate.

## Introduction

Community engagement in research has helped to shape the way research is conducted over the past decade. In particular, researchers have increasingly utilized community advisory boards (CABs) to provide feedback on their projects. One survey of 48 Center for Clinical and Translational Science (CTSA) grant awardees indicated that 89% had formed a CAB, a consultative service to researchers [1]. Providing a vehicle for members of the community to have a voice in research activities is a primary aim for CABs [2]. CABs can play a large role in the research process in several ways including assisting in recruitment, developing protocols, acting as a linkage between the investigators and the community, and voicing concerns surrounding research from a community viewpoint [ 3–6]. Despite the rise in the use of CABs for health research [7], little is known regarding the perceived impact that CABs have on the research. For example, it is not certain whether the feedback researchers received from a CAB actually shaped or impacted an investigator’s project.

To ensure that ongoing translational research conducted at Mayo Clinic Center for Clinical and Translational Science (CCaTS) respects community values and benefits community members, the Mayo Clinic established a CAB at each site in Jacksonville, Florida (formed 2008); Rochester, Minnesota (formed 2012); and Scottsdale/Phoenix Arizona (formed 2013) [8]. Each CAB is comprised of diverse community members (range of 17–25 members) representing various stakeholders in the community including patient advocates, community health care providers, public health organizations, peer scientists, and faith-based community leaders. CAB members provide a diverse perspective of local communities’ health needs and priorities, as well as a voice to ensure the research is aligned with the values and concerns of the communities. The CAB serves as a mechanism to link researchers to the local community. In this role, the CAB members identify and facilitate community partner connections and provide advice on ethical and cultural appropriateness, appropriate measurements and materials, accurate interpretation of research results, and ways to disseminate findings along with next steps to community members. During CAB meetings, members provide feedback and guidance to investigators at various stages of their research project and/or grant submission.

CAB members are provided with education and training to provide feedback and guidance in all aspects of research across the translational science continuum (e.g., developing research questions, research study design, ethical issues around research, culturally tailored dissemination methods). Education and training were provided during orientation, along with yearly refresher training, which was structured and created jointly with the CAB leadership.

Prior research conducted by the study team showed that 63% of the research topics presented across the three Mayo CTSA CABs were aligned with the health needs identified in the local community health needs assessments [8]. The current program evaluation builds on this prior work by assessing, among researchers, their appraisal of the impact of the CAB’s feedback on different phases of the research.

## Materials and Methods

### Researcher Community Advisory Board Presentations

Investigators request guidance and advice at various stages in their research project and/or grant submission. To help researchers prepare for their CAB presentation and facilitate discussion, researchers are asked to provide a brief summary of their research project (e.g., aims, target population, etc.), describe how the research will benefit the patient or community health, provide any research material to review (e.g., protocol, recruitment flyers, surveys, etc.), and provide a list of specific questions for how the CAB can assist. Presentations and discussions are approximately 30 min in length; with presentations lasting 10–15 min, leaving 15–20 min for discussion with the CAB members. Feedback is provided at the time of the presentation and in writing by the CAB coordinator approximately 3 weeks after the presentation takes place.

### Procedures

From 2014 to 2017, 34 researchers presented their research project to a Mayo Clinic CAB at Minnesota (*n* = 23), Arizona (*n* = 3), and Florida (*n*= 8). From September 2019 to November 2019, researchers were invited by email to participate in the interview in person or by phone. A second invitation was emailed at 2 weeks and a third invitation was emailed at 4 weeks. No remuneration was offered. The response rate was 50% (17/34). The remaining researchers had no response to the three email invitations (*n* = 9) or were not reachable because they were no longer at Mayo Clinic (*n*= 8). Of the 17 participants, 4 were women and 13 were men. These individuals presented to the MN (*n* = 14), AZ (*n* = 0), and FL (*n* = 3) CABs, respectively, a mean of 4 years (range: 3–5) before the interview. Two trained interviewers conducted the individual interviews in person or by phone, with an average interview duration ranging from 10 to 15 min.

### Measures

The interviewer used the Community Stakeholder Impact on Research Taxonomy measure [9], a valid, structured 7-item quantitative measure to assess domains of perceived influence that the CABs had on the research. Researchers were asked “*In what ways did the feedback you received from the CAB influence your research?*” Researchers were asked to indicate if the CAB feedback may have influenced each of the following domains: (1) pre-research (e.g., generated ideas), (2) infrastructure (e.g., budget preparation), (3) research design, (4) implementation (e.g., research recruitment and data collection), (5) analysis, (6) dissemination (e.g., peer-reviewed efforts and community), and (7) post-research (e.g., assist in formulating next steps for future studies). These seven domains of impact for community stakeholder engagement in the research were based on a taxonomy recommended for measuring community stakeholder contributions to research. Researchers were asked about the CAB feedback potentially influencing all seven domains of potential influence, regardless of whether the researcher specifically sought CAB input in these areas. We did not assess the researcher presentations on the stage of the research presented or for which stage(s) CAB members discussed and provided feedback.

For each of the seven domains selected by the researcher per above, the facilitator encouraged participants to provide open-ended feedback to provide examples, illustrations, and other responses. At the end of the interview, researchers were asked, “*Would you be willing to share your experiences with the CAB in a brief video interview?”* Detailed notes of the responses to each question were taken verbatim during the interview.

At the end of the interview, researchers who were willing to share their experience with the CAB were connected to social media staff to record the video testimonial in the upcoming 1–2 weeks. Researchers were informed about the purpose and use of the video and at the start of the recording were asked to state their name and that they approved the use of this video. The social media staff provided guidance on the researcher’s testimony by asking them to describe why they engaged the CAB and how working with the CAB impacted their research. The videos took an average of 10–20 min to record and the final videos were an average of 1–2 min in duration.

### Analysis

#### Quantitative analysis

SPSS software (version 25) was used to analyze descriptive statistics (frequencies, percentages, means, standard deviations) for the proportion of respondents who endorsed each of the seven domains of impact for the Community Stakeholder Impact on Research Taxonomy measure as well as an overall mean score with a possible range from 0 to 7 (higher scores indicate greater impact).

#### Qualitative analysis

Of the 17 participants, 12 provided at least 1 open-ended response, with a total of 29 comments analyzed. Using content analysis [10], data from the open-ended responses were coded by two authors independently employing an iterative process. Themes were generated based on consensus for each domain of influence based on feedback provided across the researchers. Any discrepancies in coding were discussed with the third author until consensus was reached.

Of the five videos created, one video testimonial was selected for illustration. The audio was transcribed verbatim to provide the narrative of the researcher’s experience.

## Results

### Perceived Impact of Community Advisory Boards on Research

The mean total Community Stakeholder Impact on Research Taxonomy measure score was 1.8 (SD=1.7, range: 0–6). The proportion of researchers reporting the CABs had influenced their research for the seven domains was 24% in pre-research, 24% infrastructure, 41% study design, 41% implementation, 6% analysis, 24% dissemination, and 18% for post-research activities.

### Researchers’ Input on How Community Advisory Boards’ Feedback Influenced the Research

#### Pre-research domain

Researchers described the impact of engaging CAB members in the pre-research stage as helping to generating ideas, identifying needs important to the community, and providing perspective to research question framing. For example, one researcher stated, “*Feedback led to doing the survey to learn if patients and family have access [to healthcare]. This formed the base of data to apply toward the grant.”* Another researcher indicated, “*They identified potential community partners and ways to implement the study intervention.”*

#### Infrastructure domain

Several researchers articulated that CAB members’ engagement in the infrastructure stage impacted the planning process and distribution of funds. One researcher shared that CAB members provided recommendations on appropriate compensation, “*They helped with barriers to inviting community with the invite letter and appropriate remuneration.”*

#### Study design domain

Engaging CAB members in the study design stage impacted how the study was conducted. This was indicated by one researcher explaining how issues were identified for addressing ethical consent and material literacy, “*They helped address privacy and asked if we planned on using different language skills.”* Another researcher explained how the CABs feedback addressed cultural appropriateness, potential stigma related to a health condition, and assessment of community comfort level with the study plans, “*The CAB suggested not using the word obesity, instead using the term healthy weight. They felt that the word obesity could be misconstrued and stigmatizing.*” In addition, several researchers described how the feedback received provided guidance to determine the way the research was applied, such as, “*We really considered the thought provided by the CAB to formulate Figure 2 in the [peer reviewed] paper”* and “*The feedback we received from the CAB members allowed us to create a more robust community engagement research plan that we could share with our research team (multisite).”*

#### Implementation domain

CAB members’ feedback influenced the implementation stage in the areas of participant recruitment strategies, identifying potential patient and community stakeholders, and with communication strategies for the intervention delivery. One researcher explained, “*The biggest advice was to go out into the community and make it family friendly; to involve the community or make them aware of the research project. Their feedback helped in the overall development the activity curriculum and in where the project took place… not at Mayo but in the community. This has made the program accessible to families and that is one of the biggest feedback we hear from parents is how nice it is to have easy access to the facility.”*

#### Analysis domain

Impact in the analysis stage was highlighted by one researcher who spoke about how CAB members’ feedback provided insight into cultural appropriate interpretations of the research results, as well as brought attention to important factors in the data that they had not considered, “*they helped with the interpretation of the result to the community and potential cofounders we might have missed.”*

#### Dissemination domain

Influence in the dissemination stage was described in the areas surrounding culturally appropriate messaging, in addition to who in the community could benefit from hearing the study results. One researcher shared, “*Their recommendations given provided insight on how to approach additional patients and community stakeholders locally, as well as to raise awareness about the impact of uterine fibroids in diverse communities*.”

#### Post-research domain

Researchers who sought input on formulating the next steps in the post-research stage found CAB members’ feedback influenced recruitment platforms (e.g., Facebook and Twitter) to reach a wider audience, identifying potential local funding resources, and new community partners to collaborate.

### Researcher Testimonials

Among the 17 researchers, 5 (29%) were willing to share their experiences in a video. This research was planning a study on women’s health and uterine fibroids. The research team had just received funding for a multisite prospective study. The researcher, study coordinator, and a co-investigator presented information at the CAB meeting. The study was in the IRB submission phase and the CAB members were asked to provide insight on study recruitment. After an initial presentation, CAB members were asked to provide details on the topics that should be covered in the recruitment letters to potential participants. The other aspect related to ensuring that the recruitment letter was culturally appropriate for diverse women. The CAB asked questions related to functional literacy in English. At the time of the study, it was only approved for participants that could read and speak in English. After the initial CAB meeting, the research coordinator asked if they could send the recruitment letter to CAB members for feedback via email. Members agreed to provide virtual feedback. This allowed for the study team to maintain contact with the CAB after the initial meeting.

The study team implemented the CAB’s suggestions into the IRB protocol and the study was approved by the IRB. A follow-up presentation was done by the research team to gain insight on how to increase minority enrollment into the same prospective study. The research team presented a community-engaged research process they planned to use. The CAB was asked to provide insight on other places, partners, and audiences to engage in this study on women’s health. The CAB suggested that the research team reach out to community organizations, external clinics, and groups that focused on diverse women. The board suggested conducting some educational efforts around women’s health to strengthen overall engagement and recruitment for the prospective study.

*“We were doing a study looking at women who were diagnosed with uterine fibroids and we wanted to find out the best ways to recruit women of minority status, mainly African American women, into research studies. The CAB provided excellent feedback in terms of thinking about our recruitment materials; they also reviewed all of our questionnaires; the information that was given to the patients in terms of the study flow, so how the study would be operationalized; and even reviewed our consent documents. This led us to create a study experience for our participants that was really reflective of the needs of the community, as well as the patients. The CAB also gave us some insights on the fact that it was very important for us to think about returning those results that we found within this clinical trial back to the local community and we are working on those efforts now.”*

## Discussion

This program evaluation provides new information on researchers’ experiences working with CABs and how this stakeholder feedback impacted the research. All researchers emphasized the CABs as supportive of their research and the input received as positive and valuable in guiding their research. Although researchers expressed positive remarks and benefits in working with the CABs, the impact of the CAB feedback assessed quantitatively had a moderate impact on the research. The quantitative impact of CAB feedback may be underestimated as researchers were asked to report on all seven domains, whereas their research presentation may have addressed only a few areas. As a baseline assessment, we aimed to assess the overall impact CABS was having on the research in these domains. These preliminary data can serve as a baseline to evaluate the implementation of strategies to enhance the impact of CAB feedback on future research.

The qualitative findings from the open-ended responses reflected new ideas or directions for the researcher to consider predominantly in the stages of study design and implementation. Major themes focused on the need to address ethical consent and material literacy, ideas for culturally tailoring and targeting recruitment strategies, identifying potential patient and community stakeholders, and suggestions for shaping communication strategies for the intervention delivery. The bidirectional communication fostered ideas to address cultural appropriateness and reduce stigma related to health condition barriers, making the research relevant and appropriate. The feedback provided researchers with a better understanding of the needs important to the community, as well as identifying potential local funding resources and community partners to collaborate.

Understanding ways in which CABs could provide a larger impact on the research is essential to enhancing CTSA. Our results suggest several directions for research to enhance the impact of CAB feedback on research. One strategy is to encourage or require researchers’ presentations to address at each of the domains when receiving institutional internal funding. Moreover, before an initial presentation, researchers could be provided with a copy of the interview domains for ideas on how CAB feedback could be useful so that they could consider these in a proactive manner. Having more touchpoints with the researcher and providing follow-up input could lead to a larger impact at more stages. Methodologically, it would be important to conduct a follow-up interview on CAB impacts more proximal to the researcher presentations. This approach may increase interview participation as some investigators had left the institution, as well as enhance recall as on average several years had lapsed since researchers included in this program evaluation had presented to the CABs.

Another strategy to increase CAB impact could be for co-chairs to meet with researchers prior to their presentations to help prepare their presentations and shape their questions. Another method is to raise awareness of CABs by fostering bidirectional communication between the researchers and community members and patient stakeholders at the early phases of the project. Additional strategies that could improve CAB feedback are showing results to the CAB members and getting their reactions to the researchers’ comments, as well as providing updates to CAB members and looking at how other medical/academic CABs operate to learn if they are doing something different.

## Strengths

Key strengths of this program evaluation are the use of a validated interview guide and mixed qualitative and quantitative methods.

## Limitations

Major limitations of this program evaluation are the small and possibly unrepresentative convenience sample, with an unequal number of interviews completed across the three CABs. Future studies could build on these preliminary data by obtaining a purposeful sample with interviews completed until data saturation is reached [11]. The timeframe between the researcher presentation and interview averaged 4 years. Multiple researchers experienced challenges to recall specific details of the CABs feedback and could only provide the domain and elements of the study that were impacted. This tended to be most prominent among the researchers who presented in 2014 and 2015. One approach to enhance impact would be to conduct the interviews with researchers within 3–6 months of presentation. The open-ended responses suggested the CAB feedback generated new ideas or ways to conduct the research. However, we did not ask researchers to comment specifically on whether CAB feedback encouraged a revision in existing thinking or addressed issues the researcher has not thought about. Future research could evaluate these features of how CABS influences the research.

## Conclusion

Despite positive experiences noted in open-ended feedback, the perceived quantitative impact of CAB feedback on the research was moderate. Qualitative findings indicate that bidirectional communication between researchers and CAB stakeholders has the capacity to make clinical and translational research more relevant and appropriate.
